# Catheter-directed thrombolysis for patients with acute lower extremity deep vein thrombosis: a meta-analysis^1^

**DOI:** 10.1590/1518-8345.2309.2990

**Published:** 2018-06-21

**Authors:** Wang Li, Zhang Chuanlin, Mu Shaoyu, Chao Hsing Yeh, Chen Liqun, Zhang Zeju

**Affiliations:** 2 MSc, RN, School of Nursing, Chongqing Medical University, Chongqing, Chongqing, China; 3 MSc, RN, The First Affiliated Hospital, Chongqing Medical University, Chongqing, Chongqing, China.; 4 Professor, School of Nursing, Chongqing Medical University, Chongqing, Chongqing, China.; 5 PhD, Professor, School of Nursing, Johns Hopkins University, Baltimore, MD, United States of America.; 6 MSc, RN, School of Nursing, Chongqing Medical and Pharmaceutical College, Chongqing, Chongqing, China.

**Keywords:** Upper Extremity Deep Vein Thrombosis, Venous Thrombosis, Efficacy, Meta-Analysis

## Abstract

**Objectives:**

To evaluate case series studies that quantitatively assess the effects of catheter-directed thrombolysis (CDT) and compare the efficacy of CDT and anticoagulation in patients with acute lower extremity deep vein thrombosis (DVT).

**Methods:**

Relevant databases, including PubMed, Embase, Cochrane, Ovid MEDLINE and Scopus, were searched through January 2017. The inclusion criteria were applied to select patients with acute lower extremity DVT treated with CDT or with anticoagulation. In the case series studies, the pooled estimates of efficacy outcomes for patency rate, complete lysis, rethrombosis and post-thrombotic syndrome (PTS) were calculated across the studies. In studies comparing CDT with anticoagulation, summary odds ratios (ORs) were calculated.

**Results:**

Twenty-five articles (six comparing CDT with anticoagulation and 19 case series) including 2254 patients met the eligibility criteria. In the case series studies, the pooled results were a patency rate of 0.87 (95% CI: 0.85-0.89), complete lysis 0.58 (95% CI: 0.40-0.75), rethrombosis 0.11 (95% CI: 0.06-0.17) and PTS 0.10 (95% CI: 0.08-0.12). Six studies comparing the efficacy outcomes of CDT and anticoagulation showed that CDT was associated with a reduction of PTS (OR 0.38, 95%CI 0.26-0.55, p<0.0001) and a higher patency rate (OR 4.76, 95%CI 2.14-10.56, p<0.0001).

**Conclusion:**

Acute lower extremity DVT patients receiving CDT were found to have a lower incidence of PTS and a higher incidence of patency rate. In our meta-analysis, CDT is shown to be an effective treatment for acute lower extremity DVT patients.

## Introduction

Deep vein thrombosis (DVT) in the lower extremities is a common vascular disease. DVT not only affects the treatment and prognosis for patients but also represents a significant clinical and economic disease burden on health care systems^1^ ^).^ The annual incidence of DVT in the leg is between 48 and 182 per 100,000 in the population^2^. As the population ages, the incidence of DVT is steadily increasing^3^. DVT can be complicated by pulmonary embolism (PE) in the short-term and, in the long-term, can cause post-thrombotic syndrome (PTS), which can adversely affect quality of life^2^.

The goals of treatment for acute lower extremity DVT are to prevent PE and reduce the incidence of PTS^4^. Conventional anticoagulant treatment is mainly aimed at the prevention of PE and recurrent DVT^5^; nevertheless, over half of DVT patients have developed some degree of PTS in the follow-up period after therapy^6^. Elastic compression stockings had also been recommended by the American College of Chest Physicians Evidence-Based Clinical Practice Guidelines for DVT patients to prevent PTS (9th edition) ^(^ ^7^. However, a meta-analysis (six random controlled trails including 1462 patients) recently showed that elastic compression stockings are not sufficient to prevent PTS^1^. Due to the limited effectiveness of anticoagulant therapy for DVT, catheter-directed thrombolysis (CDT) was developed by interventional radiologists as an invasive treatment for DVT in 1994^8^. Although CDT was suggested by the American College of Chest Physicians Antithrombotic Therapy for Venous Thromboembolism (VTE) Disease CHEST Guideline^6^ in 2016, evidence to support CDT for DVT is limited. To evaluate the evidence to support CDT for DVT, we conducted a meta-analysis.

The purpose of this meta-analysis was to (1) evaluate case series studies that quantitatively assess the effects of CDT and (2) compare the efficacy of CDT and anticoagulation in patients with acute lower extremity DVT. 

We followed the Preferred Reporting Items for Systemic Reviews and Meta-Analysis (PRISMA) statement for reporting the results of this meta-analysis^9^.

## Methods

The literature search was performed using Ovid MEDLINE (1946 to January 2017), PubMed (January 31, 2017), Embase (1974 to January 2017), Cochrane Library (1999 to2016) and Scopus (1966 to January 2017). Boolean logic was used with search terms including (“catheter-directed thrombolysis” OR “catheter-directed therapy” OR “catheter-directed treatment”) AND (“deep vein thrombosis” OR “venous thromboembolism”). Additional studies were identified from the reference lists from the selected articles. Endnote software was used to manage the citations obtained through the database search. 

Two authors (Wang and Zhang) independently established the study eligibility in the meta-analysis; any difference in the opinion about the eligibility was resolved by discussion or by consulting the corresponding author (Mu) and the research team. All abstracts were reviewed using inclusion and exclusion criteria in order to narrow the selection of studies considered for the meta-analysis. The studies had to meet the following eligibility criteria: (1) studies about CDT for the treatment of acute lower extremity DVT or studies comparing CDT plus anticoagulation with anticoagulation alone; (2) RCTs, nonrandomized comparative studies and case series studies; (3) reported the data on one or more study outcomes (PTS, complete lysis, patency rate, recurrent DVT); (4) patients were ≥18 years old; (5) sample size ≥10 patients; (6) articles were published as peer-reviewed English studies. Studies were excluded if they were (1) studies irrelevant to CDT; (2) studies reporting chronic or upper DVT; (3) studies that provided no useful data; (4) studies that were case reports or duplicate articles.

Data were extracted from all included studies by two independent reviewers. Disagreements about discrepancies were resolved by consulting the corresponding author. We extracted data about the first author’s name, publication year, study design, region, mean age of patients, the ratio of men to women, treatment method, thrombolytic agent, effectiveness outcomes (PTS, complete lysis, patency rate, recurrent DVT), the time of follow-up and method of DVT diagnosis.

Assessment of bias risk was independently performed by two investigators (Wang and Zhang). The quality of the included randomized clinical trial (RCT) studies was assessed using the Jadad scale. The quality items scored were the following: studies’ description of randomization (2 points), blinding (2 points) and attrition information (1 point). Scores ≤2 is divided into low-quality publication and ≥ 3 is divided into high-quality publication^10^. All included non-randomized comparative and case series studies were appraised by The Newcastle-Ottawa scale (NOS)^11^. The quality of a study was judged on the selection of the study groups, the comparability of the groups, and the ascertainment of the outcomes. High quality was deemed if the studies received a star in every domain.

The efficacy outcomes included the occurrence of PTS, the rate of complete lysis, the patency rate and rethrombosis.

(1) The occurrence of PTS is diagnosed by the Villalta scale including five symptoms (pain, cramps, heaviness, paresthesia, and pruritus) and six clinical signs (pretibial edema, skin induration, hyperpigmentation, redness, venous ectasia, and pain during calf compression). Each sign/symptom is rated as 0 (none), 1 (mild), 2 (moderate), or 3 (severe) and the points are summed to yield the total score: 0-4 no PTS; 5-14 mild/moderate PTS; 15 or more, severe PTS or the presence of ulcer^12^.
(2) The percentage of thrombolysis was defined as Grade I (≤50%), Grade II (50-90%), and Grade III (complete thrombolysis)^13^.
(3) The patency rate is the percentage (0-100%) of patency post treatment. Patency was defined as regained when the following findings occurred: Flow in the iliac and femoral vein, compressibility of the vein, and no functional venous obstruction^14^.
(4) Rethrombosis is defined as imaging proven DVT involving a new venous segment or a previously involved venous segment for which symptomatic and imaging improvement had been obtained in a patient with at least one prior episode of DVT^15^.

We used software Stata 12.0 (Stata Corporation, College Station, TX, USA) to perform the meta-analysis. The data on efficacy outcomes in the case series studies were pooled proportions and the data in RCT or nonrandomized comparative studies were extracted to calculate odds ratios (OR) and associated 95% confidence intervals (CIs). All meta-analyses were performed using both fixed and random effects models. Cochrane’s Q statistic and I^2^ statistics were calculated to provide information about the heterogeneity between studies. I^2^ statistics <25% was considered as low heterogeneity, and I^2^ statistics >50% was considered as high heterogeneity, according to the method suggested by Higgins and his colleagues^16^. The publication bias was tested using the Egger’s regression asymmetry test^17^ and Begg adjusted rank correlation test^18^. Additionally, we performed subgroup analyses based on thrombolytic agent and study design. Several sensitivity analyses were done to test the robustness of our findings. All statistical tests were two-tailed.

## Results

**Figure 1:**
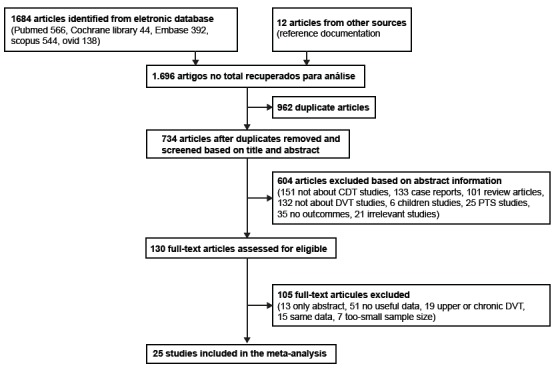
Flowchart of the study selection process.

After the database searches, 1,684 articles were retrieved, and a further 12 potential articles were identified from citations. In total, 734 unique citations were identified by our electronic searches after the deletion of duplicate publications by screening the study titles and abstracts. After applying the inclusion and exclusion criteria, 25 articles were considered for our meta-analysis, among which were 19 case series studies^19^ ^-^ ^37^ involving 1647 patients and another six studies^14^ ^,^ ^38^ ^-^ ^42^ comparing CDT with anticoagulation involving 607 patients, which fulfilled the eligibility criteria. The data abstraction process is shown in Figure 1.

Six comparison studies including 4 RCTs^14^ ^,^ ^39^ ^-^ ^41^ and 2 nonrandomized comparative studies ^38^ ^,^ ^42^, nineteen case series studies including 9 prospective studies^19^ ^-^ ^22^ ^,^ ^25^ ^-^ ^26^ ^,^ ^28^ ^,^ ^30^ ^,^ ^33^ and ten retrospective studies^23^ ^-^ ^24^ ^,^ ^27^ ^,^ ^29^ ^,^ ^31^ ^-^ ^32^ ^,^ ^34^ ^-^ ^37^ were all published in peer-reviewed journals. Except for one study^32^ that did not describe the method of DVT diagnosis, the others confirmed the presence of DVT using Duplex ultrasound or venography. When CDT was performed, rt-PA, urokinase, Alteplase or Retavase was infused. The characteristics of the included studies are summarized in Table 1.

**Table 1:** Characteristics of studies included in this meta-analysis extracted from the databases Ovid MEDLINE, 1946- 2017; PubMed, 2017; Embase, 1974 - 2017; Cochrane Library 2016; Scopus, 1960-2017, Chongqing, China, 2016-2017

Study	Design	Region	Mean age (year)	Male/Female (No)	Treatment Method (No)	Thrombolytic Agent	Outcomes	Follow up	Method Of DVT^¶^ diagnosis
Studies compared CDT* with anticoagulation									
AbuRahma et al. 2001	prospective	USA	47	21/30	CDT*+AA^†^ (18) VS AA^†^ (33)	Urokinase rtPA	major complications, PTS^‡^, patency rate	5 years	venous duplex imaging/iliofemoral phlebography
Elsharawy et al. 2002	RCT	Egypt	46	11/24	CDT*+AA^†^ (18) VS AA^†^ (17)	Streptokinase	PTS^‡^, Complete lysis	6 months	Colour duplex ascending venography
Enden et al. 2012	RCT, multicenter	Norway	52	119/70	CDT*+AA^†^ (90) VS AA^†^ (99)	alteplase	Complications, Patency rate PTS^‡^, Recurrent DVT	2 years	routine ultrasound, Or by venography or CT
Enden et al. 2009	RCT, multicenter	Norway	52	64/39	CDT*+AA^†^ (50) VS AA^†^ (53)	alteplase	iliofemoral patency, venous obstruction,	6 months	routine ultrasound, Or by venography or CT
Haig et al. 2016	RCT	Norway	52	110/66	CDT*+AA^†^ (87) VS AA^†^ (89)	alteplase	PTS^‡^, QOL	5 years	routine ultrasound, Or by venography or CT
Lee et al. 2013	Retrospective	USA	53	27/26	CDT*+AA^†^ (27) vs AA ^†^ (26)	Urokinase	Patency rate,Complications, PTS^‡^, Venous function	15months	Venography ultrasound
Case series Studies without a comparison group									
Bækgaard et al. 2010	Prospective	Denmark	29	23/78	CDT* (101)	rt-PA	Vein reflux, PTS^‡^, rethrombosis, Patency rate, mortality	6 years	ultrasonography
Bjarnason et al. 1997	Prospective	USA	47	27/50	CDT *(77)	urokinase	Complication, mortality Patency rate	5 years	Duplex ultrasound
Broholm et al. 2011	Prospective	Denmark	31	24/85	CDT *(109)	rt-PA	PTS^‡^,QOL^§^, vein reflux	6 years	ultrasound
Casella et al. 2007	Prospective	Brazil	4/14	NR^‖^	CDT *(18)	rt-PA	Complete lysis, rethrombosis, Bleeding, venous reflux	1 year	duplex-scan
Du et al. 2015	Retrospective	China	59	207/220	CDT *(427)	urokinase	Complete lysis, patency rates, PTS, Complication	2 years	ultrasound or digital subtraction angiography
Duan et al. 2015	Retrospective	China	65	49/57	CDT *(106)	urokinase	Complication, rethrombosis, Patency rate	2 years	conventional venography
Engelberger et al. 2014	Prospective	Switzerland	46	35/52	CDT* (87)	rt-PA	PTS^‡^, patency, Complication, rethrombosis, Complete lysis	1 year	Duplex sonography
Fiengo et al. 2015	Prospective	UK	35	NR^‖^	CDT* (24)	urokinase	Complete lysis, PTS^‡^, vein reflux, Complication,	2 years	Ultrasound Doppler
Jackson et al. 2005	Retrospective	USA	NR^‖^	14/14	CDT* (28)	Urokinase Retavase rt-PA	Complete lysis, patency rate, Mortality	15 months	ultrasound
Kölbel et al. 2007	Prospective	Sweden	31	11/26	CDT*+stent(37)	altplase	Complication Patency	27 months	Venography color Doppler scan
Li et al. 2015	Retrospective	China	46	93/173	CDT* (266)	Urokinase	Bleeding, complication Complete lysis	NR	computed tomography venography or ultrasound Doppler
Manninen et al. 2012	Prospective	Finland	48	26/30	CDT* (56)	Urokinase	Complication, Complete lysis, Patency, PTS^‡^, mortality	3.5 years	Ultrasound venography
Park et al. 2008	Retrospective	Korea	55	10/24	CDT* (34)	Urokinase	Complete lysis, recurrence, PTS^‡^, complication, mortality	16 months	duplex scan computed venography
Protack et al. 2007	Retrospective	USA	48	27/42	CDT* (69)	rt-PA	Rethrombolysis, complete lysis, Mortality	2.1 years	NR^‖^
Sharifi et al. 2013	Prospective	USA	52	19/14	CDT*(33)	rt-PA	complete lysis, mortality, complication	22 months	Venous duplex imaging.
Sillesen et al. 2005	Retrospective	Denmark	31	7/38	CDT* (45)	alteplase	Complication, vein reflux, rethrombosis	1 year	Doppler ultrasound
Strijkers et al. 2012	Retrospective	Germany	42	18/19	CDT*(37)	Urokinase rt-PA	Patency rate, complete lysis, complications, rethrombosis,	1 year	Duplex sonography
Warner et al. 2013	Retrospective	USA	43	9/23	CDT*+ stent (32)	Alteplase	Complication, patency rate	29 months	Venous duplex ultrasonography
Xue et al. 2014	Retrospective	China	64	25/36	CDT*+ stent(61)	Urokinase	Patency, mortality, PTS^‡^ Complication	5 years	duplex

¶DVT-deep vein thrombosis; *CDT-catheter-directed thrombolysis; †AA- anticoagulation; ‡PTS-post-thrombotic syndrome; §QOL-quality of life; ‖NR-not reported

**Figure 2:**
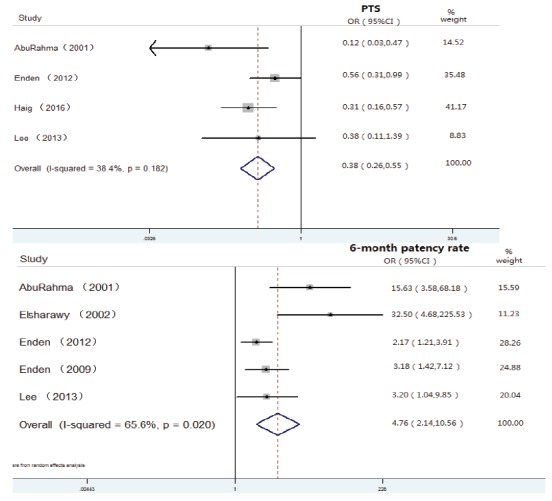
Forest plot of the pooled PTS and patency rate after CDT and CIs from CDT studies with a comparison group.

Meta-analysis of studies comparing CDT with an anticoagulation group:

(1) PTS: Four studies^38^ ^,^ ^40^ ^-^ ^42^ reported PTS data, and the pooled data showed that patients treated with CDT had a significant reduction in the occurrence of PTS (OR 0.38, 95%CI 0.26-0.55, p<0.0001) (Figure 2).
(2) Patency rate: The pooled data from five eligible studies^14^ ^,^ ^38^ ^-^ ^40^ ^,^ ^42^ suggested that the CDT group had a significantly higher 6-month patency rate than the anticoagulation group (OR 4.76, 95%CI 2.14-10.56, p<0.0001) (Figure 2).
(3) Rethrombosis: Two studies reported the results of rethrombosis^40^ ^,^ ^42^, and the pooled results showed no significant difference between the CDT and anticoagulation groups (OR 0.55, 95%CI 0.04-5.42, p>0,05).

Meta-analysis of case series studies on CDT

**Figure 3:**
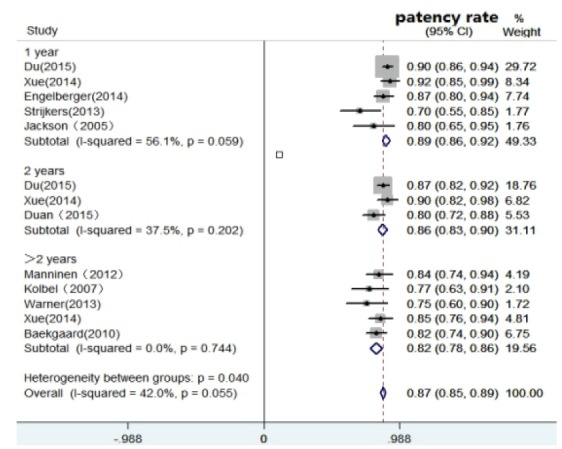
Forest plot of the pooled patency rate after CDT and CIs from reported studies according to time of follow-up.

**Figure 4:**
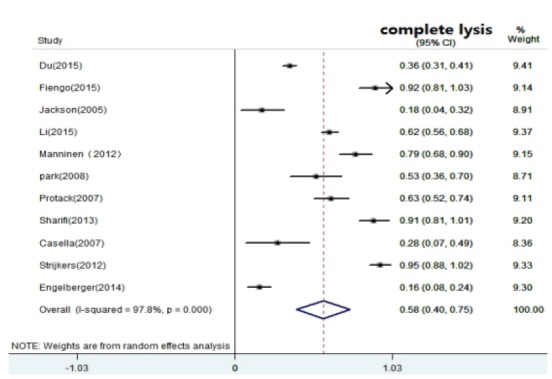
Forest plot of the pooled complete lysis rate after CDT and CIs from reported studies.

(1) PTS: 8 out of 19 (less than half) studies^19^ ^,^ ^21^ ^,^ ^23^ ^,^ ^25^ ^-^ ^26^ ^,^ ^30^ ^-^ ^31^ ^,^ ^37^ reported PTS outcomes inditcating a low incidence of PTS after CDT. The PTS rate after CDT ranged from 8% to 21%. The pooled PTS rate was 0.10 (0.08, 0.12) and I^2^ was 10.0% (p=0.353), which indicated low heterogeneity.
(2) Patency rate: Among 12 studies^19^ ^-^ ^20^ ^,^ ^23^ ^-^ ^25^ ^,^ ^27^ ^-^ ^28^ ^,^ ^30^ ^,^ ^35^ ^-^ ^37^, one study ^20^ ^)^ was eliminated because there was no total patency rate but iliac or femoral vein patency alone was reported. The patency rate after CDT ranged from 70% to 92%. Figure 3 shows that the pooled patency rate was 0.87 (0.85, 0.89), and the I^2^ was 42.0% (p=0.055), indicating moderate heterogeneity. The patency rate decreased according to the duration of follow-up, for example, 89% at 1 year, 86% at 2 years and 82% after 2 years of follow-up.
(3) Complete lysis: Eleven studies^22^ ^-^ ^23^ ^,^ ^25^ ^-^ ^27^ ^,^ ^29^ ^-^ ^33^ ^,^ ^35^ reported the rate of complete lysis, indicating the initial results of thrombolysis. The complete lysis ranged from 16% to 95% after CDT. The pooled data showed that patients treated with CDT had a moderate complete lysis 0.58 (0.40, 0.75). High heterogeneity (I^2^ =0.978, p=0.000) was detected for included studies (Figure 4).
(4) Rethrombosis: Among nine studies^19^ ^,^ ^22^ ^-^ ^23^ ^,^ ^25^ ^,^ ^30^ ^-^ ^32^ ^,^ ^34^ ^-^ ^35^, one study^34^ was excluded due to zero event of rethrombosis reported. Rethrombosis occurred in the early weeks or late years during follow-up.

The rethrombosis rate ranged from 3% to 30% after CDT. The pooled results of rethrombosis was 0.11 (0.06, 0.17), and the I^2^ was 78.4% (p=0.000), indicating high heterogeneity. 

Subgroup analyses were performed to assess the outcomes of case series studies by study design and the use of different thrombolytic agents. For the rate of patency, the results of prospective studies were slightly lower than retrospective studies. In contrast, the rate of complete lysis and PTS were slightly higher in prospective studies than retrospective studies. For the rethrombosis, it presented a bigger difference between different study designs. Subgroup analyses stratified by thrombolytic agent showed that urokinase had a better patency rate and a lower incidence of PTS. The complete lysis and rethrombosis rates were both the highest in more than 2 thrombolytic agent studies.

When assessing RCTs by Jadad score, all four RCTs had adequate descriptions for randomization and showed blinded assessment of outcomes. The information was provided in all RCTs. Therefore, the four RCTs^14^ ^,^ ^39^ ^-^ ^41^ were generally of high quality (Appendix 1). All non-RCTs and case series studies were assessed by the Newcastle-Ottawa scale; all of the 12 studies^19^ ^-^ ^23^ ^,^ ^25^ ^,^ ^28^ ^,^ ^31^ ^-^ ^32^ ^,^ ^34^ ^-^ ^35^ ^,^ ^38^ were generally of high quality. Three studies^26^ ^,^ ^29^ ^,^ ^37^ had outcomes present at the start of the study and two studies^26^ ^,^ ^33^ had no assessment of outcomes. At the same time, five studies^(24,27,30, 36,42)^ had no adequate follow-up, and one study^29^ had no report of the length of follow-up. These nine studies^24^ ^,^ ^26^ ^-^ ^27^ ^,^ ^29^ ^-^ ^30^ ^,^ ^33^ ^,^ ^36^ ^-^ ^37^ ^,^ ^42^ were generally of low quality. 

Significant publication bias was analyzed only on the patency rate of the case series studies: Begg’s Test (p=0.001), Egger’s test (p=0.001). Publication bias was not observed for complete lysis. Publication bias evaluation on other two endpoints (rethrombosis, PTS) was not detected due to the limited number of studies involved^43^. 

## Discussion

Treatment of DVT includes anticoagulant therapy, pharmacologic thrombolysis (systemic thrombolysis, flow-directed thrombolysis, catheter-directed thrombolysis), percutaneous mechanical thrombectomy, surgical thrombectomy and physical therapy (compression stockings). The previous recommendation for the treatment of acute lower extremity DVT was the use of CDT as first-line therapy^4^ ^,^ ^44^. A recent guideline of antithrombotic therapy for VTE disease still suggests that acute lower extremity DVT patients are most likely to benefit from CDT for its efficacy^6^.However, the evidence is of low quality and requires more studies to authenticate. In general, the majority of studies on CDT therapy for DVT patients were case series without control groups. Our meta-analysis including 6 comparison and 19 non-comparison studies showed that CDT was associated with a good efficacy in patients with acute lower extremity DVT. 

PTS is a chronic disorder that develops in 25-50% of patients after DVT^45^; therefore prevention of PTS is crucial. In our meta-analysis of non-comparison studies, 8 out of 19 studies recorded PTS during follow-up; less than half of the studies recorded PTS and low pooled PTS rate 0.10 (95% CI, 0.08-0.12) may indicate that CDT has a high value in preventing PTS. Already meta-analyses have evaluated the efficacy of CDT with a small number of studies. In 2012, an analysis^46^ found a significant reduction in the risk of PTS comparing CDT to systemic anticoagulation in two enrolled studies. In 2015, another analysis^47^ found the same result: a significant reduction in the risk of PTS comparing CDT plus anticoagulation to anticoagulation alone in two enrolled studies. We find the same results with four enrolled studies, showing a significant reduction of PTS with CDT compared with anticoagulation.

Our pooled analyses of non-comparison studies showed that patients with acute lower extremity DVT after CDT had a high patency rate, indicating the efficacy of CDT treatment. The pooled results of five involved comparison studies strengthen the conclusion that the CDT group has a significantly higher patency rate than the anticoagulation group. A previous meta-analysis^48^ pooling eight RCTs in China suggested that the effective rate of CDT for the treatment of acute lower extremity DVT was significantly higher than that for superficial venous thrombolysis. The reason was thought to be that thrombolytic drugs can directly act on the thrombolysis site to maximize the activation of plasminogen and effectively dissolve the thrombus. The patency rate was gradually decreased from 1 year to 2 years and more than 2 years. Existing studies showed that venous patency was directly correlated with the development of PTS^49^ ^-^ ^50^.

The pooled result of complete lysis was 0.58 (95% CI, 0.40-0.75). The Society of Interventional Radiology suggested efficacy thresholds for endovascular thrombus removal for DVT: a threshold value greater than 80% was suggested for removal of more than 50% of the thrombus^51^. However, the suggested threshold for removal of an entire thrombus is unclear. The significantly high heterogeneity observed in our paper may vary due to study designs and sample size among studies. A meta-analysis of 11 randomized anticoagulation trials showed that the residual thrombus burden after initial DVT therapy correlated strongly with the risk of recurrent venous thromboembolism (VTE)^52^.

In our meta-analysis of non-comparison studies, eight studies pooled a higher rate of rethrombosis than early recurrent thrombosis in 20 other studies according to the guidelines for the treatment of lower extremity DVT with use of endovascular thrombus removal^51^. The discrepancy may be caused by the different degree of residual thrombus burden. No evaluable difference in recurrent DVT was found between the CDT and anticoagulation-alone groups in our meta-analysis of two involved studies. The TORPEDO trial^53^ found a significant reduction in recurrent VTE comparing percutaneous endovenous intervention plus anticoagulation to anticoagulation alone. Hence, more controlled trials are needed to detect the incidence of rethrombosis for different DVT treatments.

Our subgroup analyses presented a bigger difference in rethrombosis between prospective and retrospective studies: rethrombosis in retrospective studies was 4 times higher than in prospective studies. The reasons to account for this result were as follows: in the original retrospective studies (1) laboratory hypercoagulability as a known risk factor of recurrent DVT was found in a third of all patients^31^, and (2) a delay in stent placement was considered to be the main reason for early rethrombosis^35^. Subgroup analyses stratified by thrombolytic agent showed that urokinase had better efficacy compared to two or more combined thrombolytic agents. Additionally, an existing study reported that urokinase is widely used in China for its lower cost^37^.

Several limitations should be acknowledged when interpreting the findings of our meta-analysis. First, almost half of the studies were retrospective studies, and so recall bias cannot be ruled out. Second, some data (patency rate) available for analysis were subject to publication bias because it is likely that the positive results with CDT would tend to be published. Last, only peer-reviewed English studies were included, and non-English language journals were excluded.

Nevertheless, our study also has strengths because we performed a comprehensive analysis of the efficacy results of CDT treatment by including comparison studies and non-comparison studies, which can provide available evidence about the assessment of CDT.

In conclusion, our meta-analysis indicates that the use of CDT is associated with a reduced incidence of PTS and a high patency rate. However, the efficacy of rethrombosis in DVT patients is unclear. Urokinase is the most recommended thrombolytic agent for CDT. Pharmacomechanical CDT, ultrasound-accelerated CDT and CDT combined with other assistive technologies are reasonable approaches for expanding the advantages of CDT. Finally, more well-designed RCTs to clarify and improve the efficacy and safety of CDT treatment are needed.
